# Paroxetine Triggers Inflammatory State on Zebrafish Early Embryonic Development

**DOI:** 10.3390/life15101591

**Published:** 2025-10-11

**Authors:** Elena Maria Scalisi, Agata Scalisi, Stefania Indelicato, Antonio Salvaggio, Fabiano Capparucci, Roberta Pecoraro, Maria Violetta Brundo

**Affiliations:** 1 Department of Biological, Geological and Environmental Science, University of Catania, 95124 Catania, Italy; scaliagata@gmail.com (A.S.); stefania.indelicato@phd.unict.it (S.I.); roberta.pecoraro@unict.it (R.P.); mariavioletta.brundo@unict.it (M.V.B.); 2 Experimental Zooprophylactic Institute of Sicily “A. Mirri”, 90129 Palermo, Italy; antonio.salvaggio@izssicilia.it; 3 Department of Chemical, Biological, Pharmaceutical and Environmental Science, University of Messina, 98122 Messina, Italy; fcapparucci@unime.it

**Keywords:** *D. rerio*, PRX, toxicity, ROS, acetylcholinesterase

## Abstract

Paroxetine (PRX) is a common antidepressant, also frequently used by pregnant women to treat depression and anxiety associated with pregnancy; thus, we should increase warnings about its intake. The increased presence of paroxetine in the environment raises concerns about unintended exposure to it, with consequences for embryonic development. However, the effect of PRX on early embryonic development, particularly on the embryonic brain, is still poorly studied, so this study aimed to investigate its toxicological profile on embryonated eggs of *Danio rerio*. Embryos of *D. rerio* were exposed to 1, 10, and 100 μg/L of PRX using the ZFET test. The results showed that exposure to PRX does not interfere with embryonic development but causes adverse effects in larvae, including heartbeats and an inflammatory state, with production of ROS and apoptotic cells on their head. Finally, the immunofluorescence assay for the biomarker acetylcholinesterase showed a decrease in its activity in exposed groups. Therefore, paroxetine is able to reach the nervous system during embryonic development with negative consequences.

## 1. Introduction

Antidepressions are useful for treating depression, which is the most commonly diagnosed mood disorder in the world [1]. Among the various antidepressants, there are those that can block serotonin reuptake, and for this reason, they are called “selective serotonin reuptake inhibitors (SSRIs)”. SSRIs, by limiting the reabsorption of serotonin by synaptic cells, promote its accumulation in the synaptic cleft, making it more available in the binding and activation of postsynaptic receptors [2,3]. Serotonin, or 5-hydroxytryptamine (5-HT), controls most human behaviors such as mood, appetite, sexual stimuli, memory of emotions, and anxiety at the central level, while at the peripheral level, it acts like a local hormone, especially in the gastrointestinal tract and cardiovascular and immune systems [4,5]. The synthesis of serotonin begins with the amino acid-L-tryptophan, which is an essential amino acid taken up by the body through food. Therefore, through a series of reactions, L-tryptophan will be converted into serotonin by tryptophan hydroxylase, an enzyme expressed in a small population of the raphe nucleus neurons located in the midbrain and also in enterochromaffin cells of the gastrointestinal tract [6,7]. In the central nervous system (CNS), the serotonergic system supports the production of serotonin, and, thanks to its projections, it manages to innervate all areas of the brain (the basal ganglia, limbic structures, and the cortex) [8]. It is evident that an altered regulation of serotonergic transmission causes the pathogenesis of mental illness, stress, and neurological disorders [9,10].

Clinical trials have shown the relationship between the serotonergic system and depression; therefore, many antidepressants can have the following modes of action: act directly on the serotonin receptor as 5-HT1A receptor antagonists [11,12,13], inhibit the degradation of serotonin [14], or finally, as mentioned, SSRIs can inhibit serotonin reuptake into the presynaptic neuron to promote the persistence of 5-HT in the synaptic cleft. In this way, SSRIs reduce depressive disorders by promoting an improvement in mood. As reported by the World Health Organization [15], depression is a common mental disorder, and an estimated 5.7% of adults suffer from it; women are more likely to have depression than men. The SSRIs introduced to the pharmaceutical market in the 1980s have received increasingly frequent prescriptions since the COVID-19 pandemic. Particularly, paroxetine (PRX), a derivative of phenylpiperidine, is the most effective SSRI prescribed for clinical use for the treatment of major depressive disorder (MDD), panic disorder, obsessive– compulsive disorder, social anxiety disorder (social phobia), post-traumatic stress disorder, and generalized anxiety disorders [16].

Long-term treatment of depression typically involves prolonged use of paroxetine. This is reflected in its excessive use with various side effects (nausea, headache, drowsiness, sweating, tremor, asthenia, xerostomia, insomnia, dizziness, constipation or diarrhea, and decreased appetite); furthermore, evidence in the literature highlights several consequences for men and women.

In men with normal seminal parameters, the use of paroxetine caused spermatic DNA fragmentation [17] and also changes in sperm parameters, such as decreases in motility and concentration [18], whereas in pregnant women, consequences for the fetus, such as preterm birth and low-birthweight infants during the second and third trimesters, have been observed [19]. PRX use has also led to fetal cardiovascular problems [20,21] and neonatal abstinence syndrome, including symptoms of respiratory depression, poor feeding, lethargy, nervousness [22], and risk of autism (ASD) in children, especially during the use of paroxetine in the first trimester of pregnancy [23,24,25]. This is because paroxetine taken for the mother’s needs can cross the placenta to reach the fetus; it can also be excreted in milk, resulting in fetal syndrome [26,27].

At the same time, environmental investigations have revealed an increasing presence of paroxetine in surface waters, sediments, and fish tissues [28,29,30]. The long-term presence of SSRIs on the market could also be the reason for the high cumulative concentrations of these antidepressants in sediments, in addition to their extensive consumption. In the human body, after consumption, paroxetine undergoes partial metabolism, and it is eliminated as such through feces and urine, which enter wastewater treatment plants. However, the chemical properties of paroxetine do not result in its complete removal during the wastewater treatment process; therefore, it is sometimes discharged directly into surface waters, where its concentration can reach levels up to 39.73 μg/L [31]. The persistence of paroxetine in the environment can also be determined by direct discharges of municipal, industrial, and hospital raw wastewaters [32]. In addition, improper disposal of antidepressants by consumers can contribute to increasing their presence in the environment; for example, consumers often throw antidepressants down the drain or dispose of them in the garbage [33]. Moreover, paroxetine tends to be absorbed into the sediment and could later be resuspended into the water column under certain conditions [34,35]. Consequently, antidepressants have been identified as contaminants of increasing concern (CECs) with varying concentrations in the aquatic environment, from ng/l to μg/L.

Considering all potential sources of paroxetine nowadays, paroxetine’s environmental persistence remains an open question in terms of its toxicity to neural circuits and neurogenesis at early stages of embryonic development, posing a risk to humans. Among SSRIs, paroxetine also has a higher anticholinergic activity, because it has a relatively high affinity for the M1 receptor. In patients with major depression, paroxetine not only inhibits serotonin but also partially blocks the reuptake of norepinephrine, so paroxetine may show effects similar to those of anticholinergic agents, for example, dry mouth, constipation, urinary retention, blurred vision, hypohidrosis, hypolacrimation, and confusion [36]. Considering its action on the nervous system, we evaluated the effects of paroxetine on the embryonic brain, since there are few data in the literature [37,38,39]. For this purpose, we used *Danio rerio*, called zebrafish, which is increasingly becoming an established model of vertebrate for toxicological applications [40]. The popularity of this animal model, both in embryonic and adult stages, is related to its small size, high reproducibility, rapid growth, and significant homology with humans (75% of human genes have orthologs of zebrafish) [41]. *D. rerio* can generate a large number of translucent embryos that allow the observation and study of different stages of embryonic development and can be handled without any difficulty [42]. Embryos have rapid embryonic development and immediate organogenesis; in fact, complete organs such as the heart, eyes, intestines, brain, and blood vessels are formed within 48 h of fertilization. In particular, the eyes, brain, heart, notochord, and fins are highly sensitive to disturbance [43]; therefore, we have investigated the effect of paroxetine on embryonic development in *D. rerio* and highlighted its action on the brains of embryos, because environmental exposure may occur in pregnant women and the effects of paroxetine on the fetus may be underestimated.

## 2. Materials and Methods

### 2.1. Zebrafish Embryo Collection

The embryos used for the acute toxicological test were obtained from Fish Pathology and Experimental Centre of Sicily (CISS) of the Department of Veterinary Science (University of Messina). Here, in a breeding room, the male and female adults (2:1) were placed in a hatching tank to breed, ensuring optimal conditions (light/dark cycle:14 h/10 h; 27 ± 1 °C, pH 7.2 ± 0.3, 6.00 ppm dissolved oxygen content in the water). After fertilization, the eggs were collected with Pasteur pipettes, rinsed in aquarium water at 28 °C, and then analyzed under a stereomicroscope (≥30-fold magnification) to discard those that had not been fertilized or that shown irregularities in the blastomeres or the chorion. A steel grid on the bottom of the tank prevented predation of the eggs by adults and facilitated their collection. Therefore, all fertilized eggs at the blastula stage were used to perform the zebrafish embryo toxicity test (ZFET) following the OECD guidelines [44] and protocol procedures of Pecoraro and colleagues [45].

### 2.2. Working Solution of Paroxetine

The active ingredient paroxetine was purchased from Fagron Personalizing Medicine, and it appeared as a white crystalline powder (purity 99%). The pharmacological solutions to be tested were prepared by dissolving the paroxetine powder in an embryo medium (27 ± 1 °C, pH range 6.5–8.57). Due to the increasing presence of paroxetine in surface waters, we have selected the following working solutions of 1 µg/L, 10 µg/L, and 100 µg/L, as they are close to environmental ones and have previously been studied in the literature [46,47,48]. A stock concentration of 100 mg/L was prepared and subsequently diluted in the selected solutions. All solutions were vortexed to ensure a homogeneous dispersion of the active ingredient.

### 2.3. Exposure of Zebrafish Embryos to Paroxetine

The selected eggs were placed randomly in a 24-well multi-plate; one embryo was placed in each well, filled with 2 mL of test solution. As suggested by the OECD guidelines [39], a total of 20 embryos were exposed to the working solution, while 4 embryos were exposed to the embryo medium (negative plate controls). Plates were prepared for 1 µg/L, 10 µg/L, and 100 µg/L solutions; in addition, negative control (embryos exposed to the embryo medium) and positive control (embryos exposed to 3,4-dichloroaniline at 4 mg/L) plates were prepared. Three replicates were performed for each experimental condition, negative controls, and positive controls. All plates were placed in an incubator to maintain a constant temperature (26 ± 1 °C) within each well. The exposure time was 96 h, and different toxicological endpoints were observed every 24 h with a stereomicroscope, as suggested by the OECD guidelines [44].

### 2.4. Observation of Toxicity Endpoints

Every 24 h until the end of exposure, four endpoints were evaluated using a stereomicroscope, as suggested by OECD guidelines:

- Coagulated embryos;
- Lack of somite formation;
- Non-detachment of the tail from the yolk;
- Lack of heartbeat.

In detail, coagulated embryos are the first endpoint observable 24 h after fertilization (24 hpf), indicating an acute toxic effect. Coagulated embryos are milky white and appear dark under a microscope. Somite formation occurs after 24 h, with approximately 20 somites formed in a normally developing zebrafish embryo. They allow the spontaneous movements of embryo (side-to-side contractions); thus, their absence indicates a delay in development. The detachment of the tail from the yolk sac in normal embryonic development is linked to posterior elongation of the embryonic body. The absence of tail detachment is recorded after 24, 48, 72, and 96 h. Finally, the heartbeat is visible 48 h after exposure and is recorded until the end of exposure. The absence of a heartbeat indicates the death of the embryo. Another important endpoint is the hatching of larvae, which occurs physiologically between 72 and 96 h. The hatching of larvae from the chorion results in direct exposure of the larvae to the external environment.

### 2.5. Danioscope Software: Evaluation of Heart Rate and Body Length

The transparency of the chorion allows an excellent visualization of the heartbeat and blood flow of embryos/larvae; thus, *DanioScope™* software version 1.2 (Noldus Information Technology bv Wageningen, The Netherlands), was used to assess the heart rate and body length of the larvae. After recording the toxicological endpoints, for each experimental group (control, 1 µg/L, 10 µg/L, and 100 µg/L paroxetine), a 1 min video was made using a microscope equipped with an E200 MV-R LED camera (Nikon), and at the same time, some photos were taken. The acquired files were then uploaded into the *Danioscope* software, which allowed their processing by providing quantitative data on heart rate in BPM (beats per minute). Further, the length of the larvae, obtained from the photos, was expressed in µm. All videos and photos were acquired at 72 and 96 hpf.

### 2.6. Optical Microscopy Protocol

At the end of the exposure, larvae from each experimental group (1 µg/L, 10 µg/L, and 100 µg/L) and the control group were washed in phosphate-buffered saline (PBS) for 3 min and then fixed in 4% (*w*/*v*) formaldehyde at room temperature. Therefore, some larvae were used for the optical microscopy protocol to be embedded in paraffin, while others were used to perform an immunofluorescence assay. Sabaliauskas and colleagues’ protocol [49] was followed to embed the larvae in paraffin: briefly, the larvae were dehydrated with increasing alcohol (70°, 95°, 100°), clarified in xylene, and finally embedded in paraffin (VWR-Chemicals, Radnor, PA, USA) at 60 °C with a (Thermofisher Histostar, Waltham, MA, USA) tissue processor. All paraffin blocks were sectioned to a thickness of 5 μm using a microtome, and then the histological sections were collected on microscope slides.

#### Hematoxylin–Eosin Staining

Histological sections of the larvae from each experimental group were deparaffinized in xylene, rehydrated in alcohol, and stained with Haematoxylin–Eosin (HE) (Bio-Optica, Milan, Italy) according to our standard protocol [50]. This is called histomorphological staining, which allows a detailed analysis of the morpho-structural organization of the tissues. At the end of the procedure, all stained sections were observed using an optical microscope (Set E200 Nikon, Amsterdam, The Netherlands) and the images were acquired with a camera (CMOS Nikon, Amsterdam, The Netherlands) connected to the microscope.

### 2.7. Immunofluorescence Protocol

An immunofluorescence analysis was performed to localize (in whole larvae) the biomarker of acetylcholinesterase (AChe). The procedure was based on the protocol of Pecoraro and colleagues [45]. Specifically, the mouse primary anti-acetylcholinesterase antibody (AChE) (GeneTex, 1:500, Irvine, CA, USA) and the FITC-conjugated secondary anti-mouse (1:1000 dilution) antibody were used. Images were acquired with the NIKON DS-Qi2 camera, connected to a fluorescence microscope (GeneTex). The FITC-conjugated secondary anti-mouse antibody showed a green fluorescence.

#### 2.7.1. Assessment of Reactive Oxygen Species (ROS)

Intracellular levels of reactive oxygen species (ROS) were assessed using a fluorescent probe, 2,7-dichlorodihydrofluorescein diacetate (H2DCFDA), which is a permanent ROS marker [51]. The whole larvae from each experimental group were washed twice in Hank’s Balanced Salt Solution (HBSS) buffer for 5 min each and then incubated with the H2DCFDA probe (5 µM) in the dark for 15 min at 28 °C. At the end of the incubation, the larvae were washed in HBSS, then placed on a slide [52], and observed under a fluorescence microscope (NIKON ECLIPSE Ci, Nikon Corporation, Melville, NY, USA). Photos of all experimental groups, captured by the NIKON DS-Qi2 camera (Nikon Instruments Inc., Düsseldorf, Germany), were analyzed with Image J software to assess the fluorescence intensity.

#### 2.7.2. Assessment of Cellular Apoptosis

Acridine Orange (A.O.) dye was used to visualize cell apoptosis in whole larvae. At the end of the exposure, the larvae of each experimental group were washed twice in Hank’s Balanced Salt Solution (HBSS) buffer for 5 min each and then stained with acridine orange solution (5 µg/mL) for 20 min in the dark at room temperature. Then the larvae were quickly washed with HBSS to remove excess dye and placed on a slide [53]. The slides were observed under a fluorescence microscope (NIKON ECLIPSE Ci), connected to the NIKON DS-Qi2 camera, to acquire photos for analysis with Image J software.

### 2.8. Statistical Analysis

The normal distribution of the data was verified with the Shapiro–Wilk test and, after statistical analysis was performed, using the one-way analysis of variance (ANOVA) test to compare statistically significant differences between various groups, followed by Tukey’s test. Past4Project software (Past4.03 version) was used, and the value of *p* < 0.05 (*) was considered statistically significant. All graphs were presented as mean ± standard deviation (SD). Image J software (version 1.53) was used to quantify the fluorescence intensity of the acquired images.

## 3. Results

### 3.1. Effects on Embryonic Development

**Figure 1:**
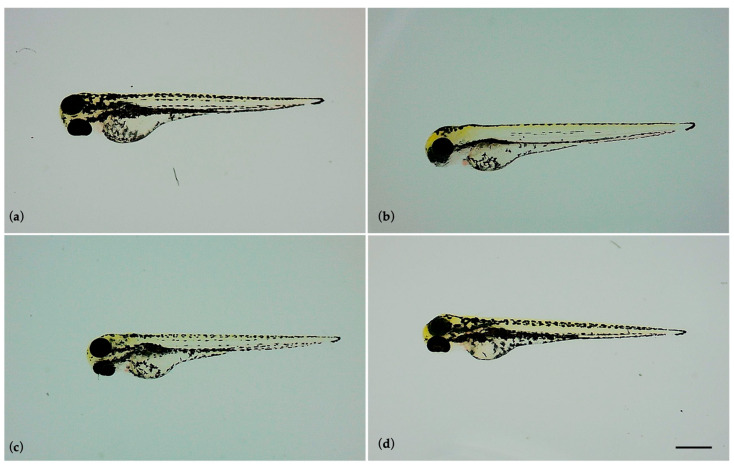
Zebrafish larvae at 96 hpf. Embryonic development was completed in all experimental groups. (**a**) Control larva, (**b**) larva exposed to 1 µg/L paroxetine, (**c**) larva exposed to 10 µg/L paroxetine, and (**d**) larva exposed to 100 µg/L paroxetine. Scale bar 560 µm.

The toxicity of paroxetine on zebrafish embryos was defined by observation of the previously described toxicity endpoints. After 24 h of exposure, a 3% of coagulated embryo was observed in the group exposed to the concentration of 100 µg/L, whereas no coagulated embryos were observed in the other experimental groups (1 and 10 µg/L) or in the control group. These percentages remained unchanged until the end of the test. Overall, exposure to paroxetine (1, 10 and 100 µg/L) did not interfere with embryonic development; in fact, the embryos of all experimental groups, as well as the control, regularly completed embryonic development with somite formation, detachment of the tail from the yolk sac, and formation of the heart and eyes. Figure 1 shows the perfectly formed larvae of all experimental groups at the end of the test (96 hpf).

At 72 h, when the larvae hatched physiologically, a decrease in hatching rate was observed in the exposed groups compared to the control, as confirmed by statistical analysis (*p* < 0.05). Specifically, the hatching rate was 60% for the control group, 38% for the 1 μg/L group, 35% for the 10 μg/L group, and 30% for the 100 μg/L group. However, it increased at the end of the test, reaching a value of 100% in the control and 1 and 10 μg/L groups, and 96% in the 100 μg/L group.

After hatching, the larvae no longer surrounded by the chorion were free to swim and thus were in direct contact with the working solutions; however, no alteration of their survival was observed. Until the end of the test, the survival of the larvae in all experimental groups remained >90%.

### 3.2. Evaluation of Heart Rate and Body Length

The larvae, and even earlier the embryos, after 48 hpf exposure, showed regular cardiac activity indicated by the presence of the heartbeat. The zebrafish heart is the first organ to develop, which, at 48 hpf, has a complete structure with an atrial and ventricular chamber, connected by the atrioventricular (AV) canal [54]. For this reason, the zebrafish heart is useful for studying cardiovascular diseases or effects induced by drugs. Thanks to the *Danioscope* software, the heart rate, expressed in BPM (beats per minute), was analyzed at 72 hpf and 96 hpf.

**Figure 2:**
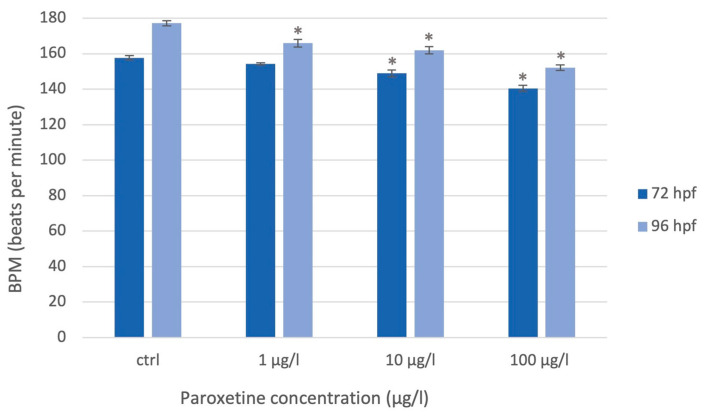
Heart rate (BPM) assessed using *Danioscope* software at 72 hpf and 96 hpf. A statistically significant decrease in BPM between the exposed groups and the control group is indicated by the asterisks (*) for a *p* < 0.05, based on the one-way ANOVA test followed by the Tukey test.

At 72 hpf, when the zebrafish’s heart rate is physiologically ±150 bpm, exposure to paroxetine caused a statistically significant decrease in heart rate for concentrations of 10 µg/L and 100 µg/L. Subsequently, as development progresses, the heart rate physiologically increases up to 180 bpm [55,56]; however, at 96 hpf, the recorded heart rate was statistically lower in all exposed groups than in the control group. *p* < 0.05 (*) indicates a statistically significant difference between the exposed groups and the control group (Figure 2). Instead, the body lengths obtained with the Danioscope software did not show a statistically significant decrease in body length for all exposed groups compared to the control group.

### 3.3. Assessment of Inflammatory State

**Figure 3:**
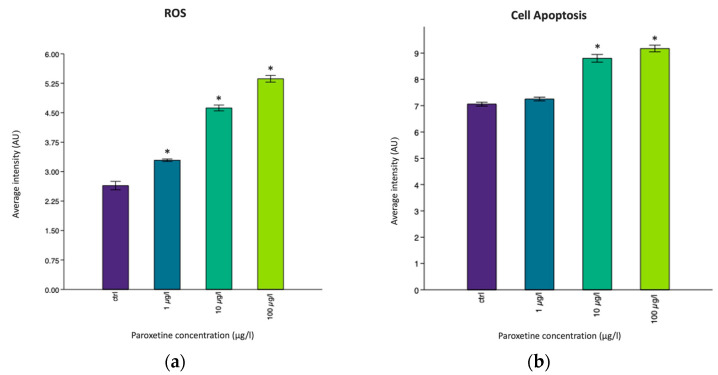
The histograms represent the average fluorescence intensity (AU) of (**a**) reactive oxygen species (ROS) and (**b**) cell apoptosis by the Acridine Orange (A.O.) dye. The asterisks (*) above the bars indicate statistically significant differences between the exposed groups and the control group (* *p* < 0.05).

The inflammatory state is often associated with the production of reactive oxygen species (ROS), which can inhibit the body’s antioxidant enzymes [57]. An oxidative stress condition, with reactive oxygen species (ROS) production and the presence of apoptotic cells, especially on the head of the larvae, was found in all exposed groups, probably because neuroactive drugs are designed to affect the central nervous system and its components. Using Image J software, the fluorescence intensity of ROS and apoptotic cells detected on the larvae was quantified, as reported in Figure 3.

**Figure 4:**
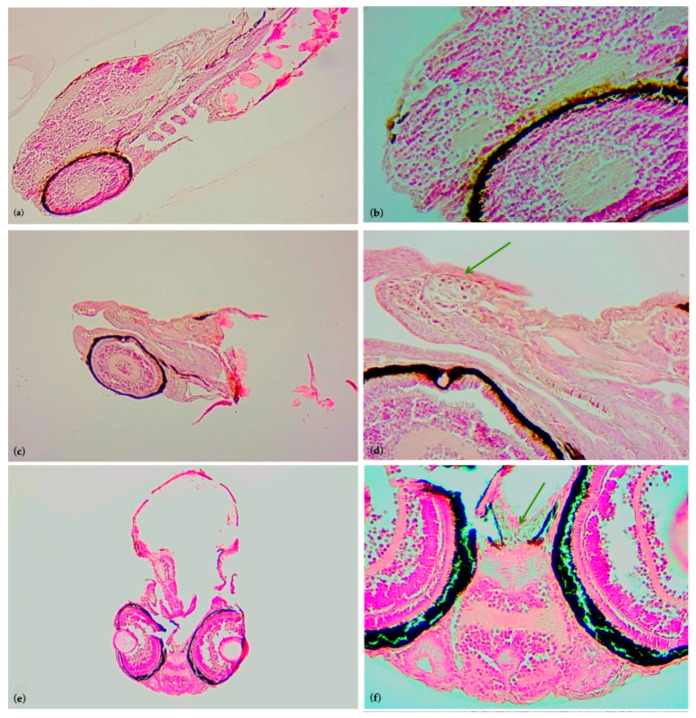
Histological sections of the larvae at 100× and 400× magnification, respectively, stained with Hematoxylin–Eosin. (**a**,**b**) Control larva, (**c**,**d**) larva exposed to 10 µg/L paroxetine, and (**e**,**f**) larva exposed to 100 µg/L paroxetine. Green arrows indicate the cellular infiltrations on the head.

**Figure 5:**
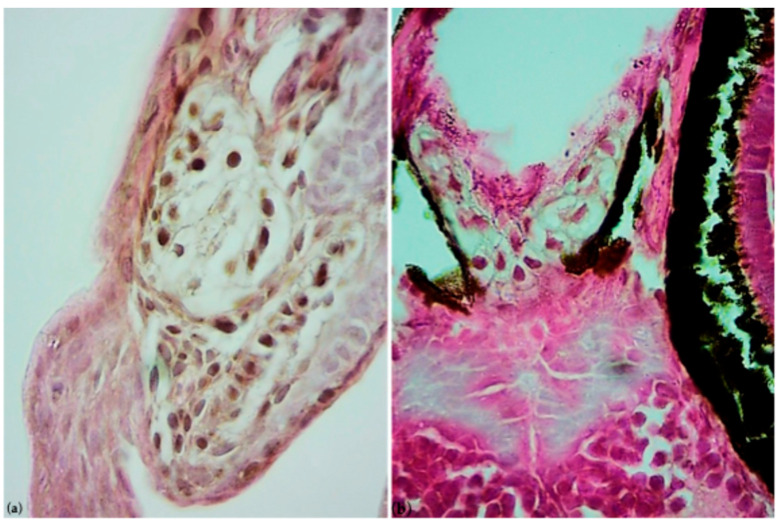
Detail of cellular infiltrations on the head of the larvae exposed to (**a**) 10 µg/L paroxetine and (**b**) 100 µg/L paroxetine. Cells appear with cytoplasmic vacuoles and eccentric nucleus, observed at 1000× magnification.

Specifically, for reactive oxygen species (ROS), a statistically significant difference (*p* < 0.05 *) was observed between all exposed groups and the control group, whereas for the apoptotic cells detected by A.O. dye, a statistically significant difference (*p* < 0.05 *) was found in the 10 µg/L and 100 µg/L groups compared to the control group. In addition, in these exposed groups, the histological investigation revealed the presence of cellular infiltration on the head of the larvae, highlighting a pool of large cells with cytoplasmic vacuoles, as shown in Figure 4 and Figure 5. These could be immune cell types, potentially macrophages that are involved in the inflammatory process, supporting the phlogosis condition induced by paroxetine.

### 3.4. Immunofluorescence Assay

**Figure 6:**
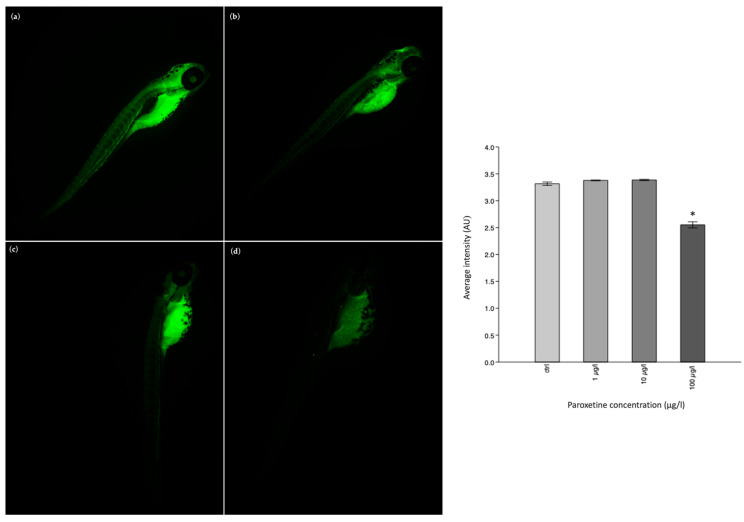
AChE antibody-staining. (**a**) Larva control, (**b**) larva exposed to 1 µg/L paroxetine, (**c**) larva exposed to 10 µg/L paroxetine, and (**d**) larva exposed to 100 µg/L paroxetine. The histogram represents the average fluorescence intensity (AU) of AChE biomarker; * *p* < 0.05.

Finally, the immunofluorescence investigation for the biomarker acetylcholinesterase (AChE) revealed a decrease in its positivity in the exposed groups compared to the control group. The fluorescence intensity of each experimental group was compared using the one-way ANOVA test. *p* < 0.05 (*) was found only in the exposed group of 100 µg/L compared to the control group (Figure 6).

## 4. Discussion

Among SSRIs, paroxetine is becoming one of the main psychotropic drugs in clinical therapy that gradually disperses into the environment, resulting in accumulation in the aquatic ecosystem [58]. Our results have shown that paroxetine does not interfere embryonic development, because from fertilization to hatching, the embryos are physiologically protected by the chorion, which acts as a barrier against environmental pollutants. But at the same time, the chorion ensures the transport of oxygen, salt ions, and nutrients needed by the embryo from the aquatic environment, also ensuring the excretion of embryonic waste products in the opposite direction [59,60]. Although no complete chemical evaluation of chorion permeability has been reported, there is widespread suspicion that the chorion affects chemical absorption, particularly in time-dependent ways. Kais and collaborators [61] showed an increase in chorion permeability at 48 hpf compared to 24 hpf; moreover, the ability of a molecule to cross the chorion depends on its chemical and physical properties. Thus, molecular weight, structure, relative bulkiness, and different substitutes can influence its absorption. Neuroactive drugs such as antidepressants, but also anxiety drugs and other psychiatric drugs, which are considered emerging environmental contaminants, pose a high environmental risk, since they are designed to cause biological effects at low doses [62]. It is known that SSRIs, even at low doses, can be pharmacologically active, and consequently, they can show biological effects in non-target organisms such as fish, algae, and invertebrates at nanogram per liter levels [63].

They are sufficiently lipophilic to be easily transported in the central nervous system, and they are also relatively resistant to degradation [64]. It has been observed that the main consequence in the absorption of these chemicals is inhibition of hatching, with consequent side effects on the embryo, such as a wavy notochord, body axis malformation, and somite defects [65]. Evidence in the literature showed that the duration of exposure and pharmacological metabolites are important factors that alter larvae behavior [46]; for example, antidepressants are among the drugs capable of significantly altering the swimming activity of larvae [46,66]. In our experimentation, an evident secondary consequence on embryonic development was the alteration of the normal physiology of the heartbeat, which we have already recorded at low concentrations, such as that of 1 µg/L selected for our experimentation. Similarly, Zhu and collaborators [67] have shown that exposure to paroxetine at concentrations higher than our experimentations, namely 5.0, 10, and 20 mg/L, caused a decrease in heart rate and blood flow; moreover, they highlighted a decrease in larvae body length. Residues of this drug in the natural environment (mainly in surface waters) can become major sources of pollution in the aquatic environment, with consequences for living organisms [68].

Even if the investigations of antidepressants have demonstrated a positive association with cardiovascular diseases [69], certainly the induction of an inflammatory state is one of the most significant toxic effects [70]. However, several studies about SSRIs have shown that they can exhibit complex and potentially opposing effects on oxidative stress linked to dosage, duration of treatment, and the individual’s underlying health status [71].

Studies report that antidepressants are able to decrease the ability to respond to stress, because they alter the hypothalamic–pituitary–adrenal (HPA) axis [72,73]; it is evident that fluoxetine and venlafaxine modify lipid peroxidation, alter the levels of protein carbonyl, and also affect antioxidant enzymes [74]. This imbalance promotes oxidation, which causes structural changes in biomolecules, such as carbohydrates, lipids, proteins, and nucleic acids, thus causing cellular alterations that can lead to high levels of inflammation and even cell death [75,76,77]. Particularly, increased oxidative stress was observed following SSRI treatment in animals that had not been subjected to stress or other types of oxidative insult. Increased lipid peroxidation and decreased antioxidant defense were noted in brain tissues of non-stressed rats following acute and chronic sertraline treatment (10 mg/kg/day, 40 mg/kg/day, 80 mg/kg/day) [78]. Pro-oxidant effects in non-stressed rats have also been reported for chronic fluoxetine treatment in hepatic tissue. In vitro studies have highlighted that SSRIs are able to increase neurological side-effects and cytotoxicity. Sertraline and paroxetine are able to induce astrocyte apoptosis, because they cause an increase in [Ca^2+^] [79,80], dysfunction of mitochondria, and activation of caspase, which lead to ROS generation [81]. Also, our in vivo results confirmed the ability of paroxetine to induce ROS generation in exposed groups. Consequently, ROS production can cause apoptosis, as reported in the astrocyte cell line [81]. The same occurred in our investigation, especially for the exposed groups of 10 µg/L and 100 µg/L, where significant fluorescence of apoptotic cells was detected. Thus, paroxetine was also able to induce cellular apoptosis, as has been demonstrated by other pollutants in several species of fish [82,83].

Moreover, according to several studies in the literature investigating the recruitment of macrophages at the site of brain injury and their different roles [84,85,86], we have highlighted the presence of large cells with phagocytic capabilities, linked to the inflammatory response, on the head of the larvae.

Several experimental and clinical studies have indicated that the combination of inflammation and oxidative stress plays a crucial role in the pathophysiology of major depression [87,88,89] and also neurodegenerative diseases, thus making paroxetine a potential risk factor for their development.

In pregnant women, it has been shown that paroxetine is able to cross the placenta, increasing the likelihood of the fetus developing neurological disorders such as Parkinson’s disease, Alzheimer’s disease, Huntington’s disease, amyotrophic lateral sclerosis (ALS), and autism spectrum disorder (ASD) when exposure to paroxetine occurs during the early stages of pregnancy [90,91,92]. In a recent study, Préta et colleagues [93], compared the human placental transfer of escitalopram, sertraline, and paroxetine; in particular, paroxetine showed an intermediate placental transfer rate of 43.4% compared to the other antidepressants investigated.

Studies have suggested negative immunoregulatory effects of antidepressants [94,95,96], and it has been shown that low-grade chronic inflammation is linked to neurological disorders. Pathological changes occurring in brain disorders reflect multifunctional changes in the immune, endocrine, and neurotransmitter circuits in the brain [96,97].

Besides serotonin, other neurotransmitters found in the neuronal circuits are dopamine, adrenaline, noradrenaline, and acetylcholine.

Acetylcholine (ACh) is a neurotransmitter involved in cognitive processes, through the activation of metabotropic muscarinic cholinergic and ionotropic nicotinic receptors.

However, thanks to the action of the acetylcholinesterase (AChE) enzyme, continuous stimulation by ACh is interrupted. The AchE enzyme acts to hydrolyze neurotransmitters into choline and acetate; thus, it is a key biomarker for synaptic transmission.

As in other vertebrates, the gene for AChE has already been identified, cloned, and functionally detected in the brain of zebrafish [98]. During vertebrate development, AChE appears before synapses are functional and its role in this context is unclear; in zebrafish, AChE is expressed in neurons before the axons reach their target [99,100]. Acetylcholinesterase is an important biomarker for many environmental contaminants in zebrafish [101,102]. For example, AchE activity was completely inhibited following exposure of embryos to diisopropylfluorophosphate (DFP), an organophosphate compound [103], and exposure to heavy metals (mercuric chloride and lead acetate) also significantly reduces the activity of acetylcholinesterase [104]. As mentioned, paroxetine is an SSRI that also has anticholinergic action, and our results suggest that in the embryonic brain, it is able to decrease the activity of the AchE enzyme. In a recent study, Sato and collaborators [92] showed adverse effects on the embryonic brain due to exposure to paroxetine; in particular, a suppression of neurogenesis was observed in the optic tectum and the dorsal telencephalon of zebrafish embryos, whereas previous studies in rats highlighted that modifications in serotonin levels in the developing brain produce negative effects on emotional behavior in adults [105,106,107]. Serotonin is crucial during brain development [108] because it plays an important role in memory and learning [109]; it has been observed that infants whose mothers were treated with paroxetine during breastfeeding developed deleterious consequences later in life, such as deficits in alertness, drowsiness, and irritability, as well as low body temperature, uncontrollable crying, and feeding and sleep disorders [96,110,111]. Therefore, neither the neuronal alteration nor oxidative stress and cardiac development caused by paroxetine should be underestimated.

## 5. Conclusions

SSRIs are the most widely used antidepressants for the treatment of major depression and anxiety disorders [112,113]. In our study we found that exposure to paroxetine, for all tested concentrations (1, 10, and 100 µg/L), does not significantly interfere with embryonic development, nor organogenesis. Despite this, exposure is related to cardiotoxicity and, in general, to oxidative stress, which leads to an inflammatory response and even cell apoptosis. Moreover, the neuronal disruption caused by paroxetine in early childhood highlights its potential role in the development of neurological disorders. Therefore, the use of paroxetine during pregnancy and breastfeeding should be limited.

## Data Availability

The original contributions presented in this study are included in the article. Further inquiries can be directed to the corresponding author.

## Abbreviations

The following abbreviations are used in this manuscript:

SSRIs	Selective serotonin reuptake inhibitors
PRX	Paroxetine
AChe	Acetylcholinesterase
